# Evaluating the impacts of school garden-based programmes on diet and nutrition-related knowledge, attitudes and practices among the school children: a systematic review

**DOI:** 10.1186/s12889-022-13587-x

**Published:** 2022-06-24

**Authors:** Chong Ling Chan, Pui Yee Tan, Yun Yun Gong

**Affiliations:** School of Food Science and Nutrition, Faculty of Environment, University of Leeds, Leeds, LS2 9JT UK

**Keywords:** School-aged children, School garden-based programmes, Nutritional knowledge, Attitudes, Food acceptability, Dietary practices, Fruits and vegetables

## Abstract

**Background:**

Previous evidence suggests that school garden-based programmes (SGBP) may be a promising yet cost-effective intervention to improve children’s knowledge, attitudes and practices (KAP) on healthy eating. This review aimed to summarise and evaluate the evidence available on the impacts of SGBP in addressing diet and nutrition-related KAP among school-aged children.

**Methods:**

Five databases including PubMed, Embase, Cochrane, Web of Science and Scopus were searched until February 2021. Randomised, non-randomised controlled and pre-post intervention studies investigating the impacts of SGBP on at least one of the outcomes of interest including diet and nutrition-related knowledge, attitudes towards fruits and vegetables (F&V), food diversity and dietary practice among school-aged children were included. Study selection and data extraction were performed by one reviewer and checked for accuracy by the other two reviewers in accordance with PRISMA guideline. Quality appraisal for studies included was assessed using American Dietetic Association Quality Criteria Checklist.

**Results:**

A total of 10,836 records were identified, and 35 studies that met the inclusion and exclusion criteria were included. This includes 25,726 students from 341 schools and 8 nurseries from 12 countries. Intervention duration ranged from 6 weeks to 4 years with 18 studies involving a varied degree of parental participation. SGBP, which majorly includes school gardening activities, cooking lessons and nutrition education, demonstrated beneficial effects on children’s nutritional knowledge, their attitudes and acceptability towards fruits and vegetables and children’s dietary practices including the actual F&V consumption and dietary diversity. However, the impacts of SGBP on such outcomes were highly influenced by various social and environmental factors including the activities/components and duration of the intervention, parental involvement, sample size, and the age of children when interventions were first introduced.

**Conclusion:**

These findings suggest that SGBP may be effective in promoting children’s nutritional knowledge, attitudes and acceptability towards vegetables, however, the impacts may vary by the type, the extent, and the length of the programmes, and other factors such as parent involvement. Future SGBP is suggested to implement using a combined multidisciplinary approach targeting the children, parents, and community to effectively promote healthy eating among the children and prevent childhood obesity.

**Supplementary Information:**

The online version contains supplementary material available at 10.1186/s12889-022-13587-x.

## Introduction

Childhood malnutrition in all forms is affecting every country in the world [1]. In the past four decades, a tenfold increase was reported in the number of obese children and adolescents aged 5 to 19 worldwide, from 11 million in 1975 to 124 million in 2016 with an addition of 213 million being classified as overweight [2]. Concerningly, childhood malnutrition is likely to persist into adulthood, which can perpetuate an ill-health cycle, increasing the health risk in their later life [3]. Suboptimal diets with poor dietary behaviour are one of the major contributing factors for both the obesity and nutritional or micronutrient deficiencies. A healthy diet, according to the World Cancer Research Fund [4] and WHO [5], is characterised by the consumption of abundant whole grains, legumes, fruits, vegetables, and nuts with a limited intake of salt, red and processed meat, sugar and fat-rich “fast food” and other processed food. Diet rich in fibre and fruits and vegetables (F&V) e.g., Mediterranean diet, has shown positive effects on tackling obesity [6–10]. Despite prominent benefits of F&V, current consumption level remains low in young people. A survey of ten European countries reported that only 23.5% of the studied children met the WHO requirement of no less than 400 g of F&V per day and more than half of the children do not consume fruits on a daily basis [11].

According to the PRECEED-PROCEED model, behavioural change occurs under the changes of its determinants [12]. In other words, having a deeper understanding of its underlying determinant is the first step in improving diet quality among children. Compelling evidence suggested that F&V consumption is driven by knowledge and awareness of, preference for and attitude towards such foods [13]. Food preferences and dietary habits are generally shaped at an early age, and they are more likely to persist into adulthood and affect our food choices in later life [14–16]. Therefore, there is a need to enhance nutritional knowledge and encourage early F&V exposure among the children, to promote their willingness to consume, acceptance and liking of F&V [17–21].

Recent evidence suggested that school garden-based programmes (SGBP) may be a promising yet cost-effective intervention to promote healthy eating habits and increase children’s F&V intake with a potential to reduce food neophobia, which is defined as the reluctance to consume novel foods [22]. School is regarded as a prime setting to shape children’s dietary behaviour whereby 20% of their daily dietary intake are obtained [23, 24]. SGBP, which enhance the circular learning environment by integrating a hands-on experimental approach, may strengthen the impact of nutrition education on children. The hands-on activities include direct gardening experiences and active involvement in designing, building, developing and maintaining the school garden with edible plants [21, 25]. Other activities may include bed preparation, seed planting, seedlings transplanting, plant growing and nurturing, and application of organic pest control [26, 27]. Growing own produces not only can increase school and/or home accessibility and availability of F&V, but also encourage children to appreciate and value garden produce [24, 25]. This may eventually increase children’s preferential selection, willingness to taste and potentially the intake of F&V. In addition to single-component SGBP interventions, multicomponent school garden-based interventions that integrate gardening with classroom curriculum, physical education, cooking session, food service, and/or with parental involvement displayed a more promising effect in promoting children’s F&V consumption and its determinants [23, 25, 28].

Despite greater potential evidence on SGBP effects towards improving knowledge, attitudes and practices (KAP) regarding diet and nutrition remain mixed. Therefore, this study aimed to systematically review the available evidence on the impacts of SGBP on diet and nutrition-related KAP among school-aged children, and to explore the key features of its effectiveness.

## Methods

### Search strategy

The search was conducted between 11th November 2020 to 6th February 2021. Five databases were used, including PubMed, Embase, Cochrane, Web of Science and Scopus for primary research articles published from year 2000. This timeframe was chosen with the aim of obtaining the most recent SGBP intervention studies. The following search terms were used: (1) school children as the targeted population: adolescent* OR boy? OR child* OR children OR girl? OR juvenil* OR kid? OR preschool* OR school* OR teen* OR youth* OR young OR “school children” OR student*; (2) school setting: school* OR nurser* OR kindergarten* OR kindergarden*; (3) garden-based interventions: garden* OR gardening OR plant* OR fruit* OR vegetable* OR “fruit vegetable*” OR “fruit growing” OR “vegetable growing” OR seed* OR tree* OR “organic agriculture” OR “organic farming” OR “organic food” OR farm; (4) outcome measures on diet and nutritional related KAP: (eating OR diet* OR food OR dietary OR nutrition OR nutritional OR fruit* OR vegetable*) AND (knowledge OR attitude OR practi?e* OR behavio?r* OR preference* OR habit* OR intake* OR consumption* OR healthy OR skill* OR pattern* OR diversity OR diverse OR perception*) OR “energy intake” OR “appetite” OR “portion size*” OR “food fussiness” OR “food neophobia”; (5) study design: “controlled trial*” OR “intervention” OR randomised OR randomized OR trial* OR “randomised controlled trial*” OR “randomized controlled trial*” OR follow-up stud* OR program evaluation*” OR “controlled before-after stud*”. Details of the search strategies used for each database are presented in the Supplementary Table 1.

### Inclusion and exclusion criteria

Studies were included if they met the following inclusion and exclusion criteria.

### Inclusion criteria

#### Population

School children and adolescents (boys and girls) aged 3–18 years old attending nursery, kindergarten, primary, secondary or high school education and special school. Children under the age of 3 and over the age of 18 would still be included as long as they were being classified as “students” or still attending nurseries, kindergarten or high schools.

#### Interventions

Studies that used school gardening, kitchen-gardening, garden curriculum or horticulture activities as primary interventions were included. Gardening activities included cultivating plants such as fruits, vegetables, shrubs, flowers and trees while gardening programmes included activities such as preparing the soil, sowing seeds, planting, weeding, watering and harvesting, hands-on learning with fruits and vegetables, education on food origins and systems, and the fresh produce’s production. Garden-related cooking and tasting activities were also included. Gardening programmes could be conducted within the school curriculum or conducted out of the lesson time such as during recess, lunchtime or after-school activities and school trips to community allotments.

#### Outcomes

Studies with a result for at least one outcome of interest were included, including examining food literacy such as diet and nutrition-related knowledge, attitudes, skills, preferences, behaviours and practices e.g., dietary diversity and F&V intake.

#### Study design

Randomised controlled trials in which individuals or clusters (classes or schools) were randomly assigned to trial arms, non-randomized controlled trials and pre-post intervention studies which examined the changes in the outcome measures at post-intervention and baseline were included. Only studies written in English were included. No restrictions were placed on the author, sample size, funding sources of study, duration of the intervention or the country where the intervention took place.

### Exclusion criteria

Garden-based interventions that did not organise by the school such as community-based gardening programmes, community youth interventions, summer holiday extra-curricular activities or clubs were excluded. Study organised by the school but occurred at the community level such community gardens, however, were included as the participants were still being regarded as “students”. Interventions with only teaching gardening related knowledge without actual hand-on gardening component were excluded. Studies that did not regard school gardening as their primary intervention or did not specify the age of participants were also excluded. Studies that only focused on describing school-based gardening programme without addressing its effects on nutritional KAP were excluded. Editorials, commentaries, opinions, review articles and observational studies such as cross-sectional studies, prospective and retrospective cohort studies were not included as well as unpublished, grey literature and ongoing studies with only preliminary findings.

### Study selection

Studies obtained from the search were uploaded to ENDNOTE (X7, Thomson Reuters). Screening and selection of studies for inclusion in this review were performed by a reviewer and the decisions were checked by the other reviewer. During the first round of screening, the title and abstract were checked for eligibility based on the inclusion and exclusion criteria. In the second round of screening, full-text articles were obtained and screened for eligibility using the same criteria. Disagreement between reviewers was resolved by discussion and by a third reviewer.

### Data extraction

A standardized data extraction form was utilized to obtain the following information, where possible: author, year of publication, journal source, source of funding, study design, year of study, country or population, sample characteristics (e.g., gender, age, socioeconomic status etc.), sample size, intervention size, control size, intervention group description (activities included in the SGBP), control group description, duration of intervention, outcome measures (e.g., indicators related to KAP around diet and nutrition) and main findings. Any disagreements were resolved through discussion with the research team.

### Strategy for data synthesis

A systematic review synthesising the qualitative evidence of school garden-based programmes was conducted. The findings on the impact of school garden-based programmes in affecting school children’s KAP around diet and nutrition were reported according to the components of the interventions via categorising them into school garden-based programmes with and without parental involvement. A meta-analysis on any of the quantitative data extracted was unable to be performed due to the heterogeneity and variation in the study design, outcome measurement and intervention component.

### Risk of bias assessment

The risk of bias of the individual studies included was independently assessed by two reviewers. Any disagreement on the risk of bias between reviewers was resolved by discussion and by a third reviewer when necessary. The risk of bias of the individual studies included was assessed using the Academy of Nutrition and Dietetics, Quality Criteria Checklist [29]. The 10 questions focus on (1) how clear the research question was; (2) selection of participants; (3) randomization/ group comparability; (4) description of withdrawals; (5) how the blinding was; (6) whether study procedures were described clearly; (7) whether the outcomes were clearly defined; (8) were appropriate statistical analyses applied; (9) did the results support the conclusion; (10) funding or sponsorship bias. To be rated positive, each of the criteria 2, 3, 6 and 7 must be met and the majority of 10 criteria overall. Any of criteria 2, 3, 6 and 7 not being met resulted in a neutral rating. If most criteria (i.e., more than 6 of them) were not met, the article would have a negative rating.

## Result

**Fig. 1:**
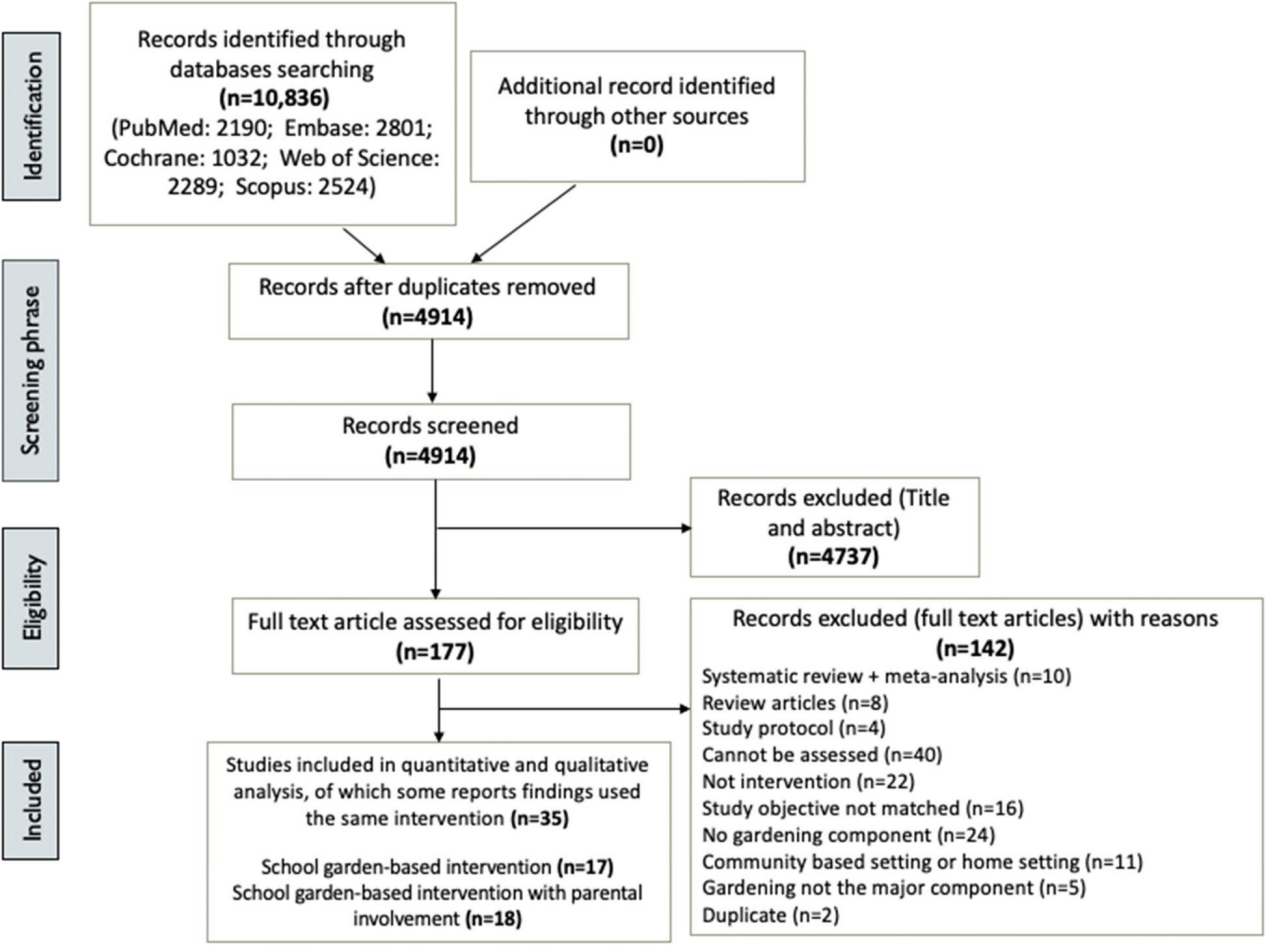
Flowchart of identification and selection of studies in accordance with PRISMA guidelines

The search from literature yielded a total of 10,836 potentially relevant articles from 5 databases (Fig. 1). After removing duplicates, 4,914 records remained. Those articles were screened for title and abstract for eligibility, resulting in 4,737 records being excluded. The full text of the remaining 177 records was assessed and examined. Using the same criteria, a total of 142 records were excluded. Thus, a total of 35 records were included in this review.

### Study characteristics

In total, 25,726 school children recruited from 341 schools and 8 nursery centres from 12 different countries were included in this review. Most of the studies were mainly reported from the populations of the United States (*n* = 18). Six studies were conducted in low- and middle-income countries (LMICs) (Nepal, Bhutan, Burkina Faso and Brazil) and 29 studies were conducted in developed countries (United States, Australia, United Kingdom, Portugal, Canada, South Korea, Netherlands and Belgium). Sample size ranged from 1 to 49 schools and 44 to 4300 participants, with more than 80% of the included studies recruited more than 100 participants (*n* = 29/35). Participants’ age ranged from 2 to 19 years old, with the majority from the age of 8–12 years old. Duration of intervention ranged from 6 weeks to 4 years (mean ± SD: 10 ± 11 months) and integrated school gardening intervention activities included outdoor or indoor classroom gardening (e.g., Earthbox gardening); harvesting lessons; cooking lessons and experimental kitchen activities utilising harvests; taste tests; nutrition-related education on food cultivation, healthy living skills, agriculture and nutrition science; physical education; healthy F&V snack program; poster, poem and nutrition and vegetable charts displays on school boards, meat-free Monday, using locally source produce in school meals and market days to sell produce from the garden and local farmers’ market visit. Outcomes of each study varied, but the majority primarily focused on the changes in children's KAP on food consumption (particularly F&V).

### Quality appraisal of included studies

**Fig. 2:**
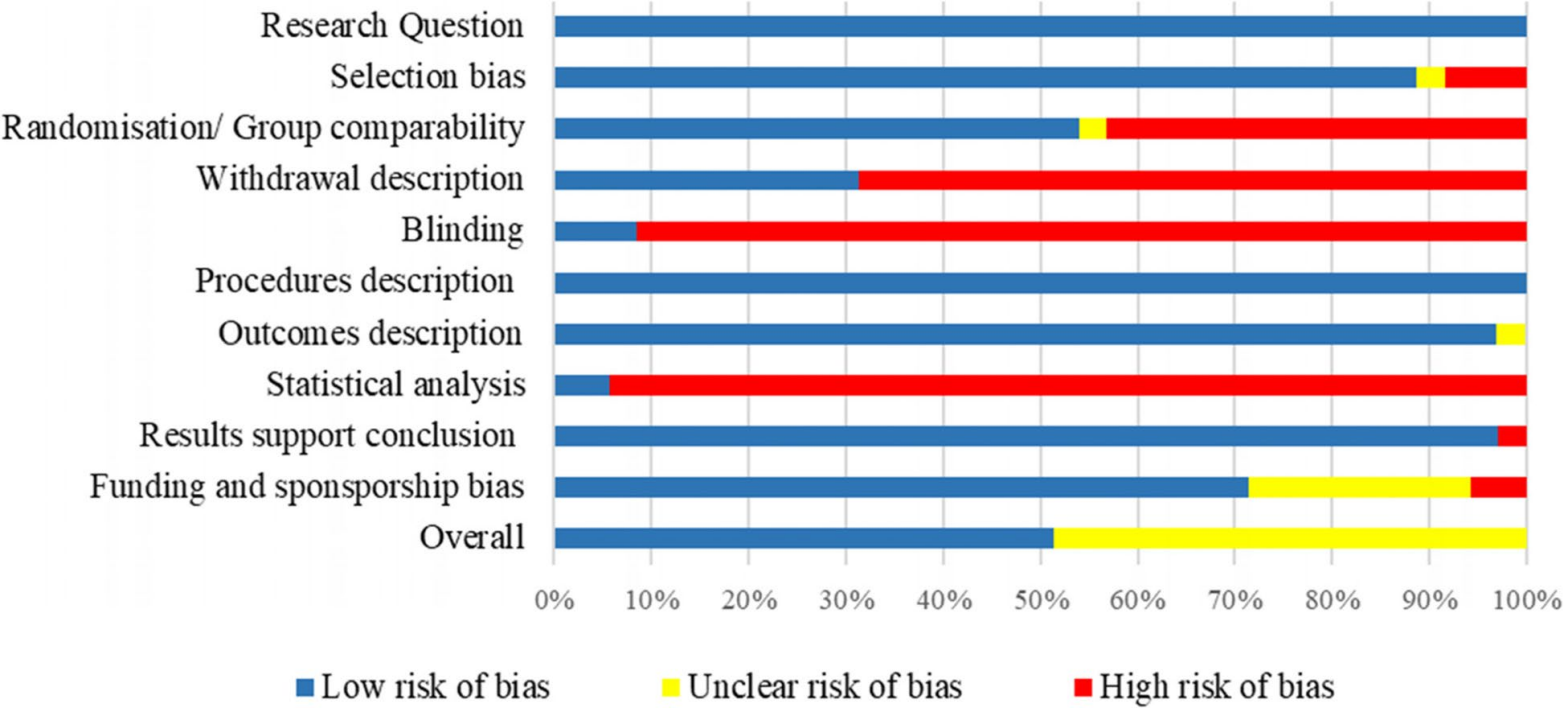
Quality rating of included studies using the Quality Criteria Checklist from Academy of Nutrition and Dietetics

The quality appraisal of the studies included is reported in Fig. 2. Almost half of the studies included had a low risk of bias with the remaining rated unclear risk (neutral). No study included had a high risk of bias. Categories that were commonly rated as weak (e.g., with more than half of the studies rated a high risk of bias) were statistical analysis, blinding and withdrawal description. Most of the studies (*n* = 33/35) failed to apply appropriate statistical analysis, studies rated as low risk of bias in this category were able to address the confounding factors as well as the application of intention to treat analysis. Majority of the studies (*n* = 32/35) failed to describe the allocation concealment or blinding of researchers, participants, or data collectors. In addition, a large proportion of studies did not describe the method of handling withdrawals (*n* = 24/35), including the follow-up method and withdrawal reasons. Detailed quality appraisal of each study is reported in Supplementary Table 2.

### Description of the included studies

A total of 35 studies have been included, and the characteristics of each intervention study are reported in Table 1. There were 18 out of the 35 studies in which the intervention included parental involvement. In this review, level of parental involvement differed between studies, ranging from students gardening with parents; student and family cooking events; parent gardening, home gardening, maintenance of school garden, school visit invitation to receive a brief of school gardening project, end-of-programme celebration invitation, take-home materials (e.g., “Family Stories” booklet and recipe cards) and parent newsletter (considered as weak parental component or low activity intensity). The main findings of the impacts of SGBP on intervention outcomes and the study quality are reported in Table 2.

**Table 1:** Summary of the included SGBP interventions (*n* = 35)

Author (Year) and location	Duration/ study design	Sample size	Sample characteristics	Parental involvement	Intervention group	Comparison or control group	Outcomes: measurement tools
Davis et al., (2021) USA [30]	1 school year/cluster RCT	16 schools (*n* = 3,135)	8–11 years old	Low^2^ 9 monthly parent lessons: 1 hour gardening, nutrition and cooking lessons – The parent curriculum also included the following topics; importance of family eating, healthy shopping, and increasing home available and access of healthy foods.	Garden Leadership Committee formation, student gardening, nutrition, and cooking lessons (either a garden taste-test (7 lessons) or a cooking activity), 9 monthly parent lessons (*n* = 1,412)	Compare: delayed intervention (*n* = 1,723)	**1) F&V and sugar sweetened beverage (SSB) consumption:** 2015 School Physical Activity and Nutrition dietary screener **2) Food and meal choice behaviours, self-efficacy to cook and/or prepare F&V and gardening, willingness to try and preferences for F&V, cooking and gardening attitudes, nutrition and gardening knowledge, and child food security**: questionnaire
Barnard et al., (2020) USA [31]	4 years/pre-post study	4 schools (*n* = 4,300)	2–19 years old	High^1^ Student and family cooking events	School Gardens and Classroom Lessons (*n* = 172 lessons), Student and Family Cooking Events, Carrot Camp (*n* = 206), Sprout Scouts (*n* = 52)	No control group (*n* = 0)	**1) Knowledge, attitudes, and awareness of the programme:** teacher survey **2) Parent/caregiver knowledge and attitudes related to the programme and its potential impact on children:** parent/caregiver survey **3) F&V consumption:**parent/caregiver survey
Kim et al., (2020) South Korea [32]	12 weeks/pre-post-test experimental design	2 schools (*n* = 202)	Average age: 11.6 (± 1.5) years old	–	Gardening, nutritional education, and cooking activities utilizing harvests (*n* = 202)	No control group (*n* = 0)	**1) Self-efficacy:** dietary self-efficacy questionnaire **2) Outcome expectancies for V consumption:** outcome expectation questionnaire **3) Food neophobia:** 10-item food neophobia scale **4) V preference:** list of representative vegetables from the Dietary Reference Intakes for Koreans 2015 **5) Nutrition and gardening knowledge:** questionnaire based on Korean elementary textbooks **6) V intake:** dietary record sheet
Schreinemachers et al., (2020) Nepal [33]	1 year/cluster RCT	30 schools (*n* = 779)	8–12 years old	High^1^ Home gardening	Consisted of a physical garden for hands-on experience in vegetable growing and nutrition education following a booklet with 23 weekly learning modules; children’s caregivers additionally received support to improve their home gardens (Children: 438; parents 437)	Control: no intervention (child: 436; parents 436)	**1) V intake**: 24-hour recall **2) Food and nutrition knowledge**: 15 MCQ **3) Agricultural knowledge**: 14 photos of common garden pests and beneficial insects **4) Liking for V:** 15 photos of V and recording their liking **5) Snack choices, food practise:** questionnaire
Shrestha et al., (2020) Nepal [34]	5 months/cluster RCT	12 schools (*n* = 682)	8–17 years old	–	School gardening programme (*n* = 172)	Compare: school gardening programme with complementary WASH, health and nutrition interventions (*n* = 197) Control: no intervention (*n* = 313)	**1) Dietary intake:** FFQ and 24-hour recall questionnaire **2) Nutrition knowledge:** questionnaire
van den Berg et al., (2020) USA [35]	6 months/ non-RCT	28 low-income schools (*n* = 1,326)	8–9 years old (42% Hispanic; 78% free/reduced lunch).	Low^2^ LEGE: gardens built with parents, took home recipe card and family stories. WAT! Program: family engagement pieces (bonus miles form), end-of-program celebration, weekly English and Spanish newsletters featuring both healthy physical and eating tips.	(Learn! Grow! Eat! Go! [LGEG]) – school garden & school curriculum (*n* = 347)	Compare 1: Physical activity (PA) intervention (Walk Across Texas [WAT!]) (*n* = 336) Compare 2: both gardening and PA intervention (combined) (*n* = 358) Control: delayed intervention (*n* = 285)	**1) V preference, nutrition knowledge, gardening with parents, previous day V consumption:** student surveys **2) Home V availability**: parent surveys
Khan et al., (2019) UK [36]	1 school year/mix method study – randomised controlled	1 school (*n* = 60)	9–10 years old	–	Gardening intervention & a Meat-Free Monday session, physical activity & knowledge of nutrition (*n* = 30)	Compare: delayed intervention (*n* = 30)	**1) Attitude towards, frequency of and preference for eating F&V:** self-report questionnaire **2) Experiences of gardening outdoors, attitudes to eating F&V**: focus group interview
Landry et al., (2019) USA [37]	12 weeks/RCT	4 schools (*n* = 290)	Low income, primarily Hispanic/ Latino, 8–11 years old	–	LA Sprouts: cooking and nutrition curriculum & gardening curriculum (*n* = 160)	Compare: delayed intervention (*n* = 130)	**1) Dietary intake:** 2007 Block Kids Food Screeners (adapted from the Block Kids 2004 FFQ) **2) Self-efficacy to cook F&V**: adapted questionnaire from Baranowski et al., 2000 **3) Motivation to cook and garden F&V:** motivation for Health Behaving from the Treatment and Self-Regulation Questionnaire
Massarani et al., (2019) Rio de Janeiro [38]	3 years/pre-post study	1 school (*n* = 89)	11–12 years old adolescent athletes (14–15 years old at follow up)	Low^2^ Building of school garden and experimental kitchen with the direct involvement of parents; maintenance of the garden (organisation and planning of the school garden); attend the semi-annual meeting	School gardening & experimental kitchen activities & health promotion class (*n* = 89)	No control group	**1) Dietary intake:** FFQ of 12 food items
Nele Huys et al., (2019) Ghent [39]	9 weeks/non-randomised controlled	17 schools (*n* = 551)	10–12 years old	–	Gardening activity: sowing, taking care of and harvesting vegetables; nutrition education in classroom (*n* = 312)	Control: no intervention (*n* = 239)	**1) Children’s V intake and determinants (knowledge and awareness):** questionnaires **2) Program evaluation:** process evaluation questionnaire (teacher and children)
Schreinemachers et al., (2019) Burkina Faso [40]	1 year/ cluster RCT	30 schools (*n* = 1,760)	8–14 years old	High^1^ Decided together what vegetables to grow in the school garden; helped to prepare the school garden and to fence it with locally available materials; helped to find water in the dry season and helped with land preparation and fencing.	School gardening; complementary agriculture, nutrition and WASH education; local farmers and other community members in school garden (2014: *n* = 500; 2015: *n* = 400)	Control: no intervention (2014: *n* = 500; 2015 *n* = 400)	**1) F&V preferences and liking:** rating for 12 V & 10 snack choices **2) F&V intake:** 24-hour recall **3) F&V awareness:** give the correct names of 20 common F&V from colour photos **4) Food, nutrition and WASH knowledge:** test adjusted from Parmer et al., 2009 & Oldewage-Theron and Egal, 2010 **5) Agriculture knowledge:** photo test
Leuven et al., (2018) Netherlands [41]	7 months/non-randomised controlled	3 schools (*n* = 150)	10–12 years old	–	Garden and nutritionbased classroom lessons, 15 outdoor gardening lessons, and 1 harvesting and cooking lesson Short term (*n* = 106) Long term (*n* = 52)	Control: no intervention (*n* = 65)	**1) Capability to identify V, preference for those V, and the opinion regarding V, gardening, and outdoor activity:** questionnaire
Taylor et al., (2018) USA [42]	1 year/RCT	2 schools (*n* = 294)	9–10 years old	Low^2^ Parent newsletters	Inquiry-based, garden-enhanced nutrition curriculum, in-class cooking demonstrations, take-home activities, family newsletters, a health fair, and school site-specific wellness committees (*n* = 161)	Compare: delayed intervention (*n* = 133)	**1) Dietary intake:** digital images of students’ lunch trays
Wells et al., (2018) USA [43]	2 years/group RCT	46 schools (*n* = 2,768)	7–11 years old low-income children (8–12 years old in second year intervention)	–	School gardening activities and nutrition education (*n* = 1,491)	Control: no intervention (*n* = 1,277)	**1) F&V availability at home**: modified version of Baranowski’s GEM-FJV Availability assessment
Gatto et al., (2017) USA [44]	12 weeks/ RCT	4 schools (*n* = 375)	Low income, primarily Hispanic/ Latino, 8–11 years old	Low^2^ Bimonthly cooking/nutrition and gardening classes offered to the parents	LA Sprouts: 45-min interactive cooking/nutrition lesson & 45-min gardening lesson (*n* = 172)	Compare: delayed intervention (*n* = 147)	**1) Dietary intake:** 2007 Block Kids Food Screeners (adapted from the Block Kids 2004 FFQ)
Lee et al., (2017) USA [45]	6 weeks (12 one-hour sessions)/pre-post study	6 centres (*n* = 89)	3–5 years old	Low^2^ 3 newsletters (health benefits of adequate F&V intake, strategies for improving home intake); encouraged to complete activities with children – selecting a favourite recipe for a class recipe book	Lessons include songs, games, and interactive learning activities involving garden maintenance and taste tests (*n* = 89)	No control group	**1) Dietary intake:** non-consecutive 3-day food records from parents **2) F&V Availability (Home):** the F&V Home Availability questionnaire
Schreinemachers et al., (2017) Bhutan [27]	1 year/ cluster RCT	18 schools (*n* = 517)	9–15 years old	High^1^ Cultivation of vegetables with children; provided advice, gardening tools and other materials and advised schoolteachers on crops and varieties to grow. Teachers visited the parents at home and encouraged home gardening	School garden; weekly lessons in gardening, nutrition, and water, sanitation and hygiene (WASH); promotion activities: poster displays, poem displays on school boards, songs, nutrition charts, vegetable charts, pledges (2014: no data; 2015: *n* = 260)	Control: no intervention (2014: no data; 2015: *n* = 265)	**1) F&V preferences and liking:** rating for 12 V + 10 snack choices **2) F&V intake:** 24-hour recall **3) F&V awareness:** give the correct names of 20 common F&V from colour photos **4) Food, nutrition and WASH knowledge:** Parmer et al., 2009 & Oldewage-Theron and Egal, 2010 **5) Agriculture knowledge:** photo test
Schreinemachers et al., (2017) Nepal [26]	1 year/ cluster RCT	30 schools (*n* = 1,370)	10–15 years old, low income		A school garden, gardening, nutrition and WASH education and promotional materials for children and parents (poster display, distribution of handouts about nutritious food and hand washing) (2014: *n* = 429; 2015: *n* = 369)	Control: no intervention (2014: *n* = 846; 2015: *n* = 416)	
Davis et al., (2016) USA [46]	12 weeks/ RCT	4 schools (*n* = 304)	Low income, primarily Hispanic/ Latino, 8–11 years old	–	LA Sprouts participants: weekly 45-minute interactive cooking and nutrition education lesson, 45-minute interactive gardening lesson, and visits to a local farmers’ market 4 times during intervention (*n* = 167)	Compare: delayed intervention (*n* = 137)	**1) F&V preferences and identification; self-efficacy to eat, cook and garden F&V; nutrition & gardening knowledge, attitudes about cooking and gardening & current home gardening practices; willingness to try F&V:** questionnaire **2) Motivation to eat, cook F&V & gardening**: Treatment and Self-Regulation Questionnaire
Duncan et al., (2015) UK [47]	12 weeks/non-randomised controlled	2 schools (*n* = 77)	4–11 years old	–	Theory-based intervention – a school garden, cooking lessons, exploring plants and growth in science and literacy (*n* = 46)	Control: no intervention (*n* = 31)	**1) F&V consumption:** measures of the TPB related to F&V consumption **2) F&V consumption behaviour**: Day in the Life Questionnaire (DILQ)
Hanbazaza et al., (2015) Canada [21]	2 school years/pre-post study	1 school (*n* = 116)	6–12 years old	–	Classroom gardening (Earth Box container gardening) & a healthy F&V snack program (*n* = 66)	No control group	**1) Children’s knowledge of F&V**: asking children to write down 5 V&F that they knew **2) F&V preferences and home consumption:** an adapted questionnaire
Sharma et al., (2015) USA [48]	8 weeks/ pre-post study	2 centres (*n* = 103)	3–5 years old	Low^2^ Children took their plants home to share with their families to encourage dialogue with parents; invite parent to End-of-program celebration	8 PLANT Garden lessons (teacher-led) with hands-on activities emphasizing gardening and nutrition (*n* = 103)	No control group	**1) Preference and willingness to try F&Vs among pre-schoolers:** pre − post self-reported parent surveys
Spears-Lanoix et al., (2015) USA [49]	5 months/ pre-post study	1 school (*n* = 44)	8–9 years old	High^1^ Gardening together, snacks and meals together, dinners eaten together, and doing physical activity together.	JMG: youth horticulture classroom curriculum – building a class garden, growing seven V, tasting and rating each V, raw, and participating in V recipe tasting	Compare: WAT (PA intervention) – family bonus miles, waling Bingo, and class activity breaks (Children: *n* = 44, parents: *n* = 34)	**1) Food availability:** parent survey **2) V preference and consumption:** student self-reported questionnaire **3) Nutrition and health knowledge:** questions about healthy living
Wells et al., (2015) USA [50]	2-years (40 lessons)/RCT	49 schools (*n* = 3,061)	6–12 years old	–	Nutrition and garden-based lessons & gardening activities (*n* = 1,622)	Control: no intervention (*n* = 1,439)	**1) Science knowledge:** 7-item MCQ selected from the University of Missouri (UM) ‘Eating from the Garden Curriculum’ survey
Bontrager Yoder et al., (2014) USA [3]	1 year/quasi-experimental baseline and follow-up assessments	9 schools (*n* = 1,117)	8–11 years old	–	Farm to School programme: Harvest of the Month, school garden, locally sourced produce in school meals & classroom lessons (*n* = 1,117)	No control group	**1) Knowledge of food, nutrition, and agriculture; attitudes toward trying FV; perception/self-efficacy for eating healthfully, and preference for, exposure of and willingness to try F&V:** questionnaire **2) Dietary intake:** FFQ, lunch tray photo observation
Cotter et al., (2013) Portugal [51]	6 months/cluster RCT	1 school (*n* = 155)	10–12 years old	–	Lessons (dangers of high salt intake), gardening activities and collection of herbs for salt substitute at home (*n* = 58)	Compare: weekly lessons about the dangers of high salt intake (*n* = 47) Control: no intervention (*n* = 34)	**1) Estimated salt intake:** derived from 24-h urinary collection (1 mEq/24 h sodium 1/4 0.058 g per day salt)
Gibbs et al., (2013) Australia [52]	2.5 years/ pre-post study	12 schools (*n* = 764)	8–12 years old	Low^2^ Did not specify	Gardening class and kitchen class (*n* = 764)	No control group	**1) Willingness to try new foods, knowledge & capacity to describe foods, and healthy eating:** separate focus group discussions, parent and child questionnaire
Gatto et al., (2012) USA [53]	12 weeks/ non-RCT	1 school (*n* = 104)	Latino 9–11 years old, more than half were overweight or obese	Low^2^ Did not specify	LA Sprouts participants: weekly 45-minute interactive cooking and nutrition education lesson, 45-minute interactive gardening lesson and visits to a local farmers’ market 4 times during intervention (*n* = 34)	Compare: delayed intervention (*n* = 70)	**1) Motivation for healthy eating:** Treatment and Self-Regulation Questionnaire **2) Attitudes About, Preferences for, Perceptions, and Self-Efficacy to Eat and Cook F&V:** validated questionnaire
Jaenke et al., (2012) Australia [54]	10 weeks/pre-post study	2 schools (*n* = 127)	11–12 years old	Low^2^ 3 newsletters (health benefits of adequate F&V intake, strategies for improving home intake); encouraged to complete activities with their children – selecting a favourite recipe for a class recipe book	Nutrition education & gardening intervention (with kitchen-based activities) (*n* = 35)	Compare: nutrition education only (*n* = 35) Control: no intervention (*n* = 57)	**1) Food preference, willingness to taste**: questionnaire +five-item food preference assessment tool **2) F&V intake**: 2 repeat 24-hour recalls
Davis et al., (2011) USA [55]	12 weeks/ non-RCT	1 school (*n* = 104)	Latino 9–11 years old, more than half were overweight or obese	Low^2^ 3 separate 60-minute parental nutrition and gardening classes	LA Sprouts participants: weekly 45-minute interactive cooking and nutrition education lesson, 45-minute interactive gardening lesson and visits to a local farmers’ market 4 times during intervention (*n* = 34)	Compare: delayed intervention (*n* = 70)	**1) Dietary intake:** 2007 Block Kids Food Screeners (adapted from the Block Kids 2004 FFQ)
Ratcliffe et al., (2011) USA [22]	13 weeks/non-randomised controlled	3 schools (*n* = 320)	11–13 years old	–	Hands-on gardening & garden-based learning activities integrated into science lesson (*n* = 170)	Compare: only garden-based sessions integrated into science class (*n* = 150)	**1) Knowledge, attitude and behaviour:** Garden Vegetables Frequency Questionnaire (GVFQ) and the Taste Test; **2) V consumption**: 24-hour recall
Morgan et al., (2010) Australia [56]	10 weeks/non-RCT	2 schools (*n* = 127)	11–12 years old	Low^2^ 3 newsletters (health benefits of adequate F&V intake, strategies for improving home intake); encouraged to complete activities with their children – selecting a favourite recipe for a class recipe book	Nutrition education & gardening intervention (with kitchen-based activities) (*n* = 35)	Compare: nutrition education only (*n* = 35) Control: no intervention (*n* = 57)	**1) F&V intake:** 24 hr. recall **2) V preference:** ‘taste and rate’ methods developed by Birch and Sullivan **3) F&V knowledge:** questionnaire used in US ‘Gimme 5’ intervention **4) Quality of school life:** the quality of school life (QoSL) instrument “
Parmer et al., (2009) USA [57]	28 weeks/ non-randomised controlled	1 school (*n* = 115)	7–8 years old	–	Nutrition education & gardening intervention (*n* = 39)	Compare: nutrition education only (*n* = 37) Control: no intervention (*n* = 39)	**1) F&V intake**: F&V survey; lunchroom observation **2) F&V preference:** F&V preference questionnaire, lunchroom observation **3) Nutrition knowledge:** questionnaire
Somerset et al., (2009) Australia [58]	12 months/ intervention trial	1 school (*n* = 252)	9–13 years old in a low socio-economic area	–	Introduction of a school-based food garden, (*n* = 130)	Compare: historical control design (*n* = 132)	**1) Attitudes towards F&V:** the attitudes questionnaire **2) Nutrition knowledge:** F&V identification survey
McAleese et al., (2007) USA [59]	12 weeks/non-randomised controlled	3 schools (*n* = 99)	10–13 years old	–	Garden-based activities & nutrition education (*n* = 45)	Compare: nutrition education only (*n* = 25) Control: no intervention (*n* = 25)	**1) Dietary intake: **three 24-hour food recall workbooks

Degree of parental involvement: ^1^High parental involvement was defined as children having direct interaction with their parents that will affect the intervention outcomes such as parents participating in gardening, cultivating and cooking sessions with children at schools, otherwise it was defined as ^2^ low parental involvement where the activities included parent newsletter distribution and take-home activities

**Table 2:** Summary on the main findings of each SGBP intervention (*n* = 35)

Author (Year) and location	Age range/ sample size	Components included in each intervention								Risk of bias^**a**^	Main findings
		Parental involvement	School gardening	Nutrition education	Cooking & kitchen lesson	Tasting section	Home gardening	Trained teacher/ specialists	Others		
Davis et al., (2021) USA [30]	8–11 years old/ 16 schools (*n* = 3,135)	v	v	v	v	v		v	Parental lessons	Low	F intake: - V intake: +* SSB intake (reduction): -
Barnard et al., (2020) USA [31]	2–19 years old/ 4 schools (*n* = 4,300)	v	v	v	v				Carrot camp; sprout scout	Unclear	Willingness to try F: - Willingness to try V: - F intake: - V intake: - Willing to discuss what have learnt at home: -
Kim et al., (2020) South Korea [32]	Average age: 11.6 (± 1.5) years old/ 2 schools (*n* = 202)		v	v	v			v		Unclear	Gardening knowledge: +*** Nutrition knowledge: +*** Preference for V: +*** V intake: +*** Willingness to try new food/ reduction in Food neophobia: +*** Dietary self-efficacy: +*** Outcome expectancies: +***
Schreinemachers et al., (2020) Nepal [33]	8–12 years old/ 30 schools (*n* = 779)	v	v	v			v	v		Low	Food and nutritional knowledge: - Agriculture knowledge: - Attitude towards V: +* Healthy food practise: +*** V intake (Oct-Dec): - V intake (Jan-Mar): +* V intake (Apr-Jun): -
Shrestha et al., (2020) Nepal [34]	8–17 years old/ 12 schools (*n* = 682)		v					v		Low	Nutrition knowledge: - V intake: - F availability (home): - V availability (home): -
van den Berg et al., (2020) USA [35]	8–9 years old/ 28 low-income schools (*n* = 1,326)	v	v	v				v		Low	Nutrition knowledge: +*** Preference for V: +*** V tasted: + *** V intake: - V availability (home): -
Khan et al., (2019) UK [36]	9–10 years old/ 1 school (*n* = 60)		v	v				v	Meat-Free Monday session on children’s healthy eating, physical activity	Low	Nutrition and plant science knowledge: - Attitude towards F&V consumption: - Preference for F&V: - F intake: - V intake: -
Landry et al., (2019) USA [37]	8–11 years old / 4 schools (*n* = 290)		v	v	v			v		Low	**Increase in cooking:** V intake: +* Dietary fibre intake: +** **Increase in gardening:** Dietary fibre intake: +*
Massarani et al., (2019) Rio de Janeiro [38]	11–12 years old/ 1 school (*n* = 89)	v	v	v	v			v		Unclear	F intake: - V intake: - Ultra-processed foods intake (reduction): -
Nele Huys et al., (2019) Ghent [39]	10–12 years old/ 17 schools (*n* = 551)		v	v						Low	Nutrition knowledge: +* Attitude towards F&V: - V intake: - Self-efficacy: -
Schreinemachers et al., (2019) Burkina Faso [40]	8–14 years old/ 30 schools (*n* = 1,760)	v	v	v				v	Local farmers & community members in school garden	Low	Food, nutrition & agriculture knowledge: +* Preference and attitude towards F: - Preference and attitude towards V: - F intake: - V intake: - F variety: - V variety: -
Leuven et al., (2018) Netherlands [41]	10–12 years old/ 3 schools (*n* = 150)		v	v	v					Unclear	V knowledge (short term): +*** V knowledge (long term): +*** Preference for V: +*
Taylor et al., (2018) USA [42]	9–10 years old/ 2 schools (*n* = 294)	v	v	v	v			v	Take-home activities; family newsletters; a health fair; school site-specific wellness committees	Low	F intake: - V intake: +** F variety: - V variety: +**
Wells et al., (2018) USA [43]	7–11 years old/ 46 schools (*n* = 2,768)		v	v				v		Unclear	**All children:** Low fat V availability at home: +* V availability overall: - **Younger children** V availability at home: ++** Low fat V availability at home: ++** High fat V availability at home: +* F availability at home: -
Gatto et al., (2017) USA [44]	8–11 years old/ 4 schools (*n* = 375)	v	v	v	v			v		Low	F intake: - V intake: x* Dietary fibre intake: +*
Lee et al., (2017) USA [45]	3–5 years old/ 6 centres (*n* = 89)	v	v	v	v	v		v		Unclear	F intake: - V intake: - V availability (home): - F availability (home): -
Schreinemachers et al., (2017) Bhutan [27]	9–15 years old/ 18 schools (*n* = 517)	v	v	v				v	Promotion activities: poster displays, songs, nutrition charts, vegetable charts, pledges	Low	Nutrition knowledge: - Preference towards F&V: +* F intake: - V intake: + * F variety: - V variety: -
Schreinemachers et al., (2017) Nepal [26]	10–15 years old/ 30 schools (*n* = 1,370)	v	v	v				v	Poster display, distribution of handouts about nutritious food and hand washing	Low	Food and nutritional knowledge: +*** Preference for F: +** Preference for V: +** F intake: - V intake: - F variety: - V variety: -
Davis et al., (2016) USA [46]	8–11 years old/ 4 schools (*n* = 304)		v	v	v			v	Local farmers’ market visit	Low	Vegetable identification (knowledge): +** Nutrition and gardening knowledge: +** Preference for F: x Preference for V: - Willingness to try F: x Willingness to try V: - Self-efficacy to eat F&V: - Home gardening: + **
Duncan et al., (2015) UK [47]	4–11 years old/ 2 schools (*n* = 77)		v	v	v					Unclear	Intentions***, attitudes***, norms***, and perceived behavioural control*** related to F&V intake: + F&V intake: +**
Hanbazaza et al., (2015) Canada [21]	6–12 years old/ 1 school (*n* = 116)		v					v	Healthy F&V snack programme	Unclear	F&V knowledge: - Preference for F: +** Preference for V: -* F intake (home): - V intake (home): -
Sharma et al., (2015) USA [48]	3–5 years old/ 2 centres (*n* = 103)	v	v	v				v		Unclear	Willingness to try F: +** Willingness to try V: +** F&V variety: - Eating behaviour: - F availability (home): - V availability (home): -
Spears-Lanoix et al., (2015) USA [49]	8–9 years old/ 1 school (*n* = 44)	v	v			v		v		Unclear	Nutrition knowledge: +*** Preference for V: +* Willingness to try V: - V intake: +*** Total F&V intake: - SSB intake (reduction): x Home availability (V): +*
Wells et al., (2015) USA [50]	6–12 years old/ 49 schools (*n* = 3,061)		v	v				v		Low	Scientific knowledge: + ***(yet the result was uniformly poor)
Bontrager Yoder et al., (2014) USA [3]	8–11 years old/ 9 schools (*n* = 1,117)		v	v				v	Harvest of the month, locally sourced produce in school meals	Unclear	Nutrition and agriculture knowledge: +*** Willingness to try F&V: + *** Lunch time F&V availability: +** F&V consumption among low intake: +*** Overall F&V intake: - F&V variety (school): +***
Cotter et al., (2013) Portugal [51]	10–12 years old/ 1 school (*n* = 155)		v	v						Low	Salt intake reduction: +
Gibbs et al., (2013) Australia [52]	8–12 years old/ 12 schools (*n* = 764)	v	v		v					Low	Nutrition knowledge (food description): - Preference for F&V (if grow in garden): +* Willingness to try new food: +* F intake: - V intake: -
Gatto et al., (2012) USA [53]	9–11 years old/ 1 school (*n* = 104)	v	v	v	v			v	Local farmers’ market visit	Low	**For all** Preference for F: x Preference for V: - **For obese/overweight:** Preference for F: x Preference for V: +**
Jaenke et al., (2012) Australia [54]	11–12 years old/ 2 schools (*n* = 127)	v	v	v	v					Unclear	Willingness to try V: +*** F intake: - V intake: -
Davis et al., (2011) USA [55]	9–11 years old/ 1 school (*n* = 104)	v	v	v	v			v	Local farmers’ market visit	Unclear	F intake: - V intake: - Dietary fibre intake: +*
Ratcliffe et al., (2011) USA [22]	11–13 years old/ 3 schools (*n* = 320)		v	v						Unclear	Improving recognition of V: +** Attitude towards & Preference for V: +* Willingness to try V: + *** V intake (school): + * V intake (home): - V variety: +***
Morgan et al. (2010) Australia [56]	11–12 years old/ 2 schools (*n* = 127)	v	v	v	v					Unclear	F&V knowledge: +*** Taste rating for V (preference): +*** Willingness to try V: +*** F intake: - V intake: -
Parmer et al., (2009) USA [57]	7–8 years old/ 1 school (*n* = 115)		v	v						Unclear	Nutrition knowledge: + ** Rating of tasted F&V: + ** Willingness to try F&V: - V choice: + * V intake: +*
Somerset et al., (2009) Australia [58]	9–13 years old/ 1 school (*n* = 252)		v					v	Funding of a teacher coordinator (facilitate integration of garden activities into curriculum)	Unclear	Nutrition knowledge (ability to identify F&V): +* Interest in trying new food: x Preference towards F: - Preference towards V: +* Dietary self-efficacy: -
McAleese et al., (2007) USA [59]	10–13 years old/ 3 schools (*n* = 99)		v	v						Low	F intake: ++*** V intake: ++*** Vitamin A intake: +** Vitamin C intake: ++* Dietary fibre intake: + **

v: Included

+: Positive findings

++: Positive findings with more significant findings shown (compare within the same study)

-: No change

x: Negative findings

*: *p* < 0.05; **: *p* < 0.01; ***: *p* < 0.001

^a^Risk of bias was assessed using Quality Criteria Checklist, which was rated as low, unclear or high risk of bias

### Major findings

**Fig. 3:**
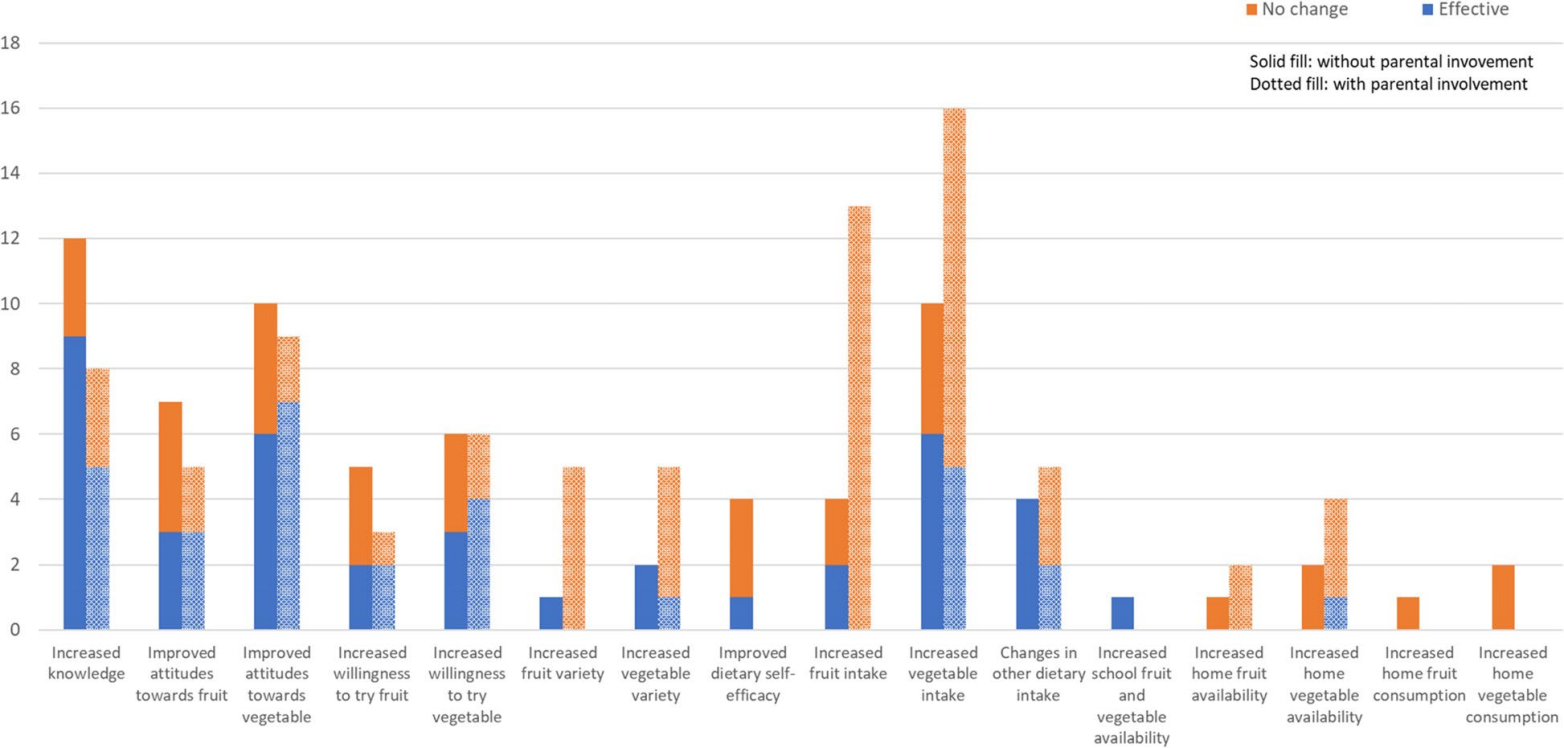
Impacts of school garden-based programmes on measured outcomes between those with and without parental involvement (*n* = 35). *Refers to food, nutrition, gardening and science-related knowledge. ** Attitudes include the concepts of preference and/ or taste ratings towards. *** Others include fibre, vitamin A & C, salts, sugary sweetened beverages and ultra-processed foods reduction

The impacts of school garden-based programmes with or without parental involvement on the children’s diet and nutritional-related knowledge, attitudes, and practices from the 35 studies included are summarised in Fig. 3. Non-significant increase is regarded as no change in terms of the effectiveness on improving the measure outcomes as reported by the studies.

### Dietary practices and food consumption

Children’s F&V intake was the most studied outcome (*n* = 26). Six out of 10 studies demonstrated SGBP without parental involvement, with a shorter intervention duration ranging from 12 weeks to 28 weeks and a smaller sample size ranging from 77 to 320 participants, resulting in a more favourable outcome on children’s vegetable intake, especially among the younger children from pre-schools and primary schools [22, 32, 37, 47, 57, 59]. Contrarily, most of the SGBP with parental involvement did not show significant improvement in children’s vegetable intake (*n* = 11/16) [26, 31, 35, 38, 40, 44, 45, 52, 54–56]. However, this may be due to the longer intervention duration ranging from 1 year to 4 years, larger sample size ranging from 89 to 4300 participants or intervening at an older age (e.g., secondary school-aged children). Similar findings were observed in children’s fruit intake, SGBP with a shorter duration (~ 12 weeks) and smaller sample size (~ 77 to 99 participants) showed better improvement in children’s fruit intake among the preschool and primary school-aged children [47, 59]. However, the majority of the SGBP with longer intervention duration (~ 1 year to 4 years) and larger sample size (~ 60 to 4300 participants), did not observe significant improvement in children’s fruit intake, regardless of the parental involvement [3, 26, 27, 30, 31, 36, 38, 40, 42, 44, 45, 52, 54–56].

Four studies reported the positive impacts of SGBP on dietary fibre, and a study showed increased intake on vitamin A and vitamin C at the post-intervention [37, 44, 55, 59]. A study conducted on the Portuguese population showed a promising effect of SGBP in reducing students’ salt intake [51], and the other three studies found no significant improvement in reducing sugar-sweetened beverages (SSB) and ultra-processed food consumption at the post-intervention [30, 38, 49]. In addition, a small number of studies investigated the impact on the variety of fruits (*n* = 6) and vegetables (*n* = 7) consumed, with the majority not able to demonstrate a significant improvement (*n* = 5/6 [26, 27, 40, 42, 48]; *n* = 4/7 [26, 27, 40, 48], respectively).

### Nutrition, gardening, agricultural and science-related knowledge

Nutrition, gardening, agricultural and science-related knowledge was the second most studied outcome (*n* = 20). Fourteen out of 20 studies reported that SGBP with or without parental involvement demonstrated significant improvement in children’s nutritional knowledge at the post-intervention, especially those shorter SGBP interventions (less than a year) integrating with classroom education and intervening at a younger age (6 to 15 years old) [3, 22, 26, 32, 35, 39–41, 46, 49, 50, 56–58]. It is worth highlighting that high sample size variability has been observed in those studies that have reported changes in children’s nutritional knowledge.

### Attitudes and behaviours towards fruits and vegetables

Two thirds of the reported studies showed significant improvement in children’s attitudes and behaviours towards vegetables at post-intervention (*n* = 13/19) [22, 26, 27, 32, 33, 35, 41, 47, 49, 52, 56–58]. Parental involvement in SGBP seems to produce better improvement in children’s attitudes towards vegetables, especially those with shorter intervention duration ranging from 12 weeks to 1 year, regardless of the sample size and the children’s age group (*n* = 7/9) [26, 27, 33, 35, 49, 52, 56]. Similar findings were observed on children’s attitudes towards fruits, the majority of the SGBP with parental involvement reported improvement on children’s attitudes towards fruits (*n* = 3/5) [26, 27, 52] compared to those without parental involvement (*n* = 3/7) [21, 47, 57]. In addition, parental involvement in SBGP seems to exert beneficial effects on improving children’s willingness to consume F&V, especially when intervened at a younger age (aged 3–12 years old) with an intervention duration ranging from 8 weeks to 2.5 years (*n* = 4/6) [48, 52, 54, 56].

### Other outcomes of interest

Four studies reported on the dietary self-efficacy in children, which refers to children’s self-belief in their food knowledge level and the self-confidence to purchase, plan, prepare and cook food, as well as to make appropriate food decisions to achieve higher nutritional value [60]. Most of the studies reported no significant improvement in dietary self-efficacy (*n* = 3/4) [39, 46, 58].

Eight studies reported the impact of SGBP on home F&V availability and consumption [21, 22, 34, 35, 43, 45, 48, 49]. Only one study from the US reported positive findings on home vegetable availability, with the remaining failing to demonstrate significant improvement [43]. In addition, one study from the US investigated the effect on school F&V availability reported a positive finding on improving school F&V availability [3].

## Discussion

### Are SGBP effective in improving diet-related knowledge, attitudes and practices among school children?

Despite the proposal of the knowledge, attitudes and behaviour communication model by Contento et al., 1992 which suggested a linear positive association between the three components that potentially influence the “practice” of such behaviour, recent research argued that such a relationship is far more reciprocal and dynamic [61, 62]. It is thus essential to understand the impacts of SGBP in addressing such association to maximise its intervention effect towards improving children F&V intake to alleviate childhood malnutrition. In this review, the findings generated from the 35 studies included indicated that school garden-based programmes were effective in increasing diet and nutritional knowledge, as well as promoting positive attitudes and behaviours (acceptability) towards vegetables among the school children, however, most of the studies reported no significant in their dietary practices such as F&V consumption and dietary diversity.

### Positive impacts on food, nutrition, gardening and science-related knowledge

In this review, SGBP have shown promising effects in improving children’s knowledge of food, nutrition, gardening and science (Fig. 2). Acquisition of knowledge is the basis for behavioural change [63]. Active participation in school gardening activities in combination with in-class food and nutrition curriculum has strengthened not only children’s horticulture skills but also children’s declarative knowledge (what is a healthy diet), procedural knowledge (how to achieve a healthy diet) and conditional knowledge (when and why healthy diet) [41, 61, 64]. Children thus have a higher potential and ability to make better and healthier food choices. In addition, children have demonstrated a greater ability to identify unfamiliar food, as less typical vegetables are intentionally emphasized in class during the intervention and incorporated into cooking activities and recipes [37]. The “seed to mouth” nature of the cooking and gardening programme, where children eat what they have grown in the school garden, also explains why children are more likely to recognize the types of crop they have consumed [37].

### Positive impacts on promoting attitudes and acceptability towards foods

SGBP may encourage children’s attitudes and acceptability to consume new food and reduce food neophobia via increasing their F&V exposure. Food neophobia refers to the reluctance to eat and try novel food, which often acts as a barrier to promote F&V preference and consumption among children and contribute to the development of unhealthful food habits [65–67]. In this review, SGBP were successful in improving children’s willingness to consume F&V. The direct involvements in growing and cooking own garden produces were associated with an increase in children self-reported willingness to consume new food [52, 56]. Additionally, the nature of the kitchen gardening programme, where children are encouraged to freely taste and share self-prepared meals with each other during kitchen class with no pressure to eat, has created a favourable social environment for children to try unfamiliar food and potentially reduce their food neophobic rate. A study conducted by Morgan et al., 2010 highlighted an increase in willingness and preference towards vegetables not only in the vegetables grown in the school garden, but also those in general, suggesting the intervention was successful in exerting influence beyond scope of the school garden, and even extended to those children did not directly expose to [56].

Interestingly, improvements were observed in children’s attitudes and acceptability towards vegetables, but not for the fruits. Previous evidence suggested that being actively involved in the food production and preparation process may exert positive influences on foods that are particularly hard to change preference towards [68, 69]. The hands-on learning experience provided by SGBP offers children regular positive exposure to vegetables. Through direct experience, for instance, crops growing, harvesting as well as food preparation, children increase familiarity with vegetables, and thus more likely to positively accept and improve taste preference towards such food [70–72]. This might also explain the insignificant intervention effect on improving children’s attitude towards fruits as the crops grown in the interventions were dominated by vegetable species, children thus had comparatively fewer opportunities to interact with fruits and thus lowering the chance to increase their acceptability towards such food.

### Limited impacts on dietary practices and food consumption

Intervention effects on improving F&V dietary intake remain inconclusive due to the mixed results generated from the studies. F&V knowledge is one of the most important determinants of their consumption [46, 73–76]. This is also supported by the social cognitive theory as augmentation in food- and nutrition-related knowledge with the acquisition of horticultural skills could increase the behavioural capacity regarding F&V intake [60, 77]. Knowledge, attitudes, taste preferences and acceptability are often being described as one of the strongest predictors for future F&V intake [21, 37, 46]. However, in this review, our finding demonstrated that SGBPs improvement in such predictors may not be sufficient to translate into an actual increase in F&V uptake.

The weak intervention effect on dietary practices found in this review denotes SGBP might fail to address other determinants that might exert a greater influence on children’s F&V consumption. This includes school and home F&V availability, parental food habits and feeding practices, the level of perceived behaviour control (e.g., ease of increasing F&V intake) and dietary self-efficacy level (i.e., self-confidence in being able to increase F&V intake) among individuals, as well as the peers’ influences [78]. Children spend most of the time learning and working as a team at school, they are more likely to follow the group perspective and consider less about their own attitudes and beliefs [79]. In other words, when the peers are more health-conscious, individuals are more likely to consume more healthy food such as F&V regardless of their own attitude towards such foods [79, 80].

Nonetheless, studies with positive findings on improving dietary practice found that SGBP was helpful in building a “personal connection” between children and the crops they have grown through direct experience on crop planting and nurturing. The “garden-grown” nature also adds an extra value to the F&V, children are thus more inclined to try and consume those healthful foods [37, 58]. An increase in school F&V availability during intervention also acts as a drive-in promoting children’s F&V consumption [22]. Moreover, SGBP can exert its influence beyond the school setting, as it is found higher varieties of vegetables not cultivated from the school garden were consumed [22]. However, the studies with positive findings may be prone to bias. For instance, the study conducted by Parmer et al., 2009 may be prone to gender bias as it was conducted predominantly in males [57]. Previous studies reported gender differences in F&V intake with the female being more likely to consume F&V and have greater perceived behavioral control and favorable attitudes towards F&V [81–84]. The positive result from McAleese et al., 2007 might be prone to measurement error, as a one-time 24-hour food recall was used [59]. The use of one-time 24-hour recall to capture food intake might fail to obtain representable data due to the varied eating habits among individuals [26, 27]. Improvements in vegetable consumption observed from Ratcliffe et al., 2011 and McAleese et al., 2007 were mainly due to the reduced intake in the control group, thus implying a small improvement in the intervention group [22, 59].

### Other measure outcomes – home food environment

In this review, there was a small number of studies that investigated the intervention impact on children’s home F&V availability (*n* = 6) and consumption (*n* = 2). Home F&V availability is known to be a crucial determinant in affecting children’s food choices. If F&V is not readily available at home, it is difficult for the children to transfer what they have learnt from the intervention into everyday life [26, 27]. However, most of the studies reported no significant impacts of SGBP on home F&V availability and consumption. The potential explanations account for the neutral effect of SGBP in modifying children’s home F&V consumption include an increase in age (lessoning effect on parental influences), family influences, unappealing presentation of F&V in home meal, low home food security, comparatively higher cost of F&V, limited accessibility to F&V within community and media influences [73, 85, 86]. With respect to home F&V availability, the authors speculated that the failure of increasing the home F&V availability may be due to the low parental participation or response rate, which subsequently hinders the children’s F&V uptake at home, as parents remain to be the main nutrition gatekeeper [35]. Since children spend a significant amount of time at home, they are more likely to enjoy and consume food that their parents enjoy, or vice versa, and prefer foods that are readily accessible and available in the home environment. Thus, it is important to improve parents’ KAP on diet and nutrition as well as the home F&V availability and accessibility to improve children’s food choices. Besides, it may be due to other determinants such as socioeconomic status and cultural influences, as well as the accessibility and availability of such foods within the neighbourhood [87–89].

### The type, the extent and the length of SGBP towards the intervention success

#### SGBP components

The nature of SGBP plays a key role in determining the success of the intervention. It is observed that all SGBP included in this review provided hand-on gardening experience, with most offered alongside nutrition education (*n* = 30), and cooking lessons (*n* = 16), which were shown to be effective in improving children’s knowledge, attitudes, and acceptability towards healthy eating practises. It is worth mentioning nutrition education is a crucial component in SGBP to improve children’s nutrition, gardening and agricultural knowledge as 12 out of 14 successful studies have integrated classroom education as part of SGBP. Other activities, including tasting sessions (*n* = 3), local farmers’ market visit (*n* = 3), local farmers and community members participating in gardening (*n* = 1), locally sourced produce included in school meals (*n* = 1) and other promotional activities such as poster display (*n* = 2), nutrition handout distribution (*n* = 1), healthy F&V snack programme (*n* = 1), Carrot camp (*n* = 1), harvest of the month (*n* = 1), meat-free Monday section (*n* = 1) and take-home activities (*n* = 1), were also investigated in some studies with unclear additional effect on improving children’s KAP. The effectiveness and the need for such components are thus questionable and worth to be further investigated. This study also investigated the effects of specialist/trained teacher delivered SGBP on improving intervention outcomes with no significant result observed.

#### Length of intervention

Previous evidence has shown school gardening interventions that succeed in enhancing nutrition knowledge lasted for a minimum of 17 weeks [39]. This review, however, reveals a minimum of 9 to 10 weeks of intervention is sufficient to observe a significant improvement in nutritional knowledge among school children aged 10–12 years old, implying a shorter intervention is as effective as a longer intervention in improving in such outcome. Similar findings were observed in the outcomes of attitudes towards vegetables (12 weeks to 1 year) and F&V intake (F: 12 weeks; V:12–28 weeks). A possible explanation is those interventions with shorter duration might have more intense effect which may not be long-lasting when intervention period lengthens. In addition, interventions that succeed in reducing children food neophobic rate in this review were mostly conducted within 5 months to 1 year, implying short intervention favours the improvement in children’s food acceptability whereas long intervention might demotivate children and potentially reduce the intervention effect, as SGBP turns from initially a “novel experience” into a “mainstream activity” [90].

#### Age

Age is one of the crucial factors in determining the effectiveness of SGBP in improving children’s food choices and dietary behaviour. In this review, it is observed that the SGBP which have been intervened at an early age may produce more favourable outcomes in improving children’s nutrition-related knowledge, willingness to consume F&V and vegetable intake. Children’s food preferences and dietary habits are generally developed and shaped early in life and may persist in adulthood, thus early childhood provides an ideal opportunity to shape healthy eating behaviours among individuals [91]. Younger children tend to have more plasticity in preferences and are more likely to accept foods that are available within their environment, which may explain why SGBP have been reported to be more successful when targeting the younger population [92].

#### Sample size

Sample size is also one of the determinants of the intervention’s success. This review revealed that the SGBP conducted in a smaller sample size produced more favourable outcomes in improving F&V intake, school interventions conducted in a smaller sample size often have a smaller teacher-student ratio with a higher teacher-student interaction, students thus receive more individualised attention and support with a higher potential to achieve better performance on intervention outcomes [93]. However, this finding was contradictory to most studies that support the usage of a larger sample size in intervention as it produces more representable, accurate and reliable results [94, 95]. Further studies are needed to explore and consolidate the relationship between sample size and intervention outcomes and understand the underlined rationale behind.

### Does parent participation benefit the SGBP?

This review reveals that parental involvement in SGBP may help to better promote children’s attitudes towards and willingness to consume F&V. Parents are known to play a fundamental role in the development and achievement of the children [96]. According to social learning theory, children learn and model from the behaviour of others through observation [97]. As children spend a substantial amount of time with parents, children’s food choice, eating behaviour and eating-related attitudes are thus hugely influenced by their parent [92, 98, 99]. Active and effective parental involvement in the SGBP, including face-to-face engagement with frequent interactions with children, provides parents more opportunities to impose positive parental modelling effects on healthy eating and enjoyment of eating F&V. Children are thus more likely to develop a preference for F&V and to make healthier food choices.

The insignificant parental effect observed in most of the studies could possibly be due to the little parental involvement element and low parents’ participation in the intervention activities. Studies conducted by Jaenke et al., 2012, Lee et al., 2017, Taylor et al., 2018, and Massarani et al., 2019 only included regular parent newsletter distribution as their main parents’ engagement activities, as newsletter could only serve as an informed purpose, parent-children’s interactions were hence limited [38, 42, 45, 54]. In addition, parents often have inflexible and overwhelming work schedules with multiple responsibilities in charge, resulting in limited time dedicated to each responsibility and hence a low parents’ participation in school activities is expected [100, 101]. Therefore, it is understandable that no significant parental effect was observed. Moreover, some of the studies did not describe the parental involvement activities in detail (e.g., the number of parents involved, and the response rate were absent), which make it difficult to assess its impacts on the effectiveness of the interventions. Future SGBP, hence, should measure the degree of parental engagement, if possible, examine what constitutes effective parental involvement and identify effective strategies to promote and maximise parental interaction with children during the intervention. To encourage more parental engagement, it requires better cooperation between the school and parents. It is suggested to schedule meetings and activities on multiple occasions to match parents’ varying schedules and be flexible in accommodating parents and families in the school programmes such as providing incentives, food or refreshments, and free transportation to minimise barriers and create an enabling environment for parents’ participation [102].

### SGBP in developed countries and LMICs

Six studies included from LMICs observed, similar to the overall findings or findings in developed countries, significant improvements in children’s preferences and attitudes towards vegetables but no significant improvement in F&V intake. Whilst improvement in food, nutrition, gardening and science-related knowledge was observed in the developed countries, it was not observed in the LMICs. The insignificant gardening effects on improving children’s knowledge and F&V consumption could be due to the inadequacy in school resources. Due to the limited number of teachers and fewer classroom materials provided, teachers from LMICs are already struggling with completing their high amount of workload. Schreinemachers et al., 2019 reported that teaching in Burkina Faso is difficult as every teacher is averagely responsible for 45 primary schoolchildren, requesting them to take on extra responsibilities and time for the implementation of an unessential gardening intervention are thus extremely hard [40]. The intervention effects might consequently be undermined as teachers are less likely to deliver the intervention programme due to the constraints of time and resources. Furthermore, the seasonal supply of vegetables also lowers the intervention effect. Due to the limited water supply in the dry season, vegetables are reported to be available for only 3 to 4 months, effect on improving children’s preference towards and intake of F&V are therefore reduced due to the limited availability and accessibility of F&V [40]. Besides, it is known that most school children from LMICs already participated in agricultural activities at home, garden-based intervention might be more appealing to those from developed countries where children lack nature experience and outdoor activities at school [40]. The parental influences on shaping children’s dietary behaviour were weak in the six studies, which is possibly due to their comparatively low education level [26, 33]. With limited knowledge and awareness of nutrition and healthy diet, parents from LMICs might be less likely to make healthier food choices for their children, family modelling impact is thus reduced.

### Strengths and limitations

One of the strengths of this review is that a comprehensive literature search was performed from five different databases to adequately identify most of the literature related to this topic, and potentially reduced the selection bias [103]. A robust review method was used, as two reviewers were involved to determine the inclusion and exclusion of studies independently. In addition, based on the Quality Criteria Checklist from the Academy of Nutrition and Dietetics (2016), none of the included studies was rated as high risk of bias, this demonstrates the high quality of the included studies.

There are a few limitations that cannot be ignored. The use of various measurement tools to assess the outcome measures increases the complexity of interpretation when comparing between studies. Heterogeneity of the intervention components, sample sizes, study designs and outcome measures between studies implied that the synthesis of the meta-analysis was not possible. Therefore, a single summary estimate of the impacts of SGBP failed to be generated [104]. In addition, result in this review is just a general sum up of findings from each study reported, age, sample size, ethnicity and study quality have not been weighted. Impacts of society-led or community-led gardening programmes have also not been explored and discussed, thus it is beyond the remit of this review to fully cover the impact and efficiency of society or community-led gardening programmes, a recent review by Ohly et al., 2016 provides a comprehensive overview of this topic [105]. Moreover, all SGBPs included were multifaceted with varying degrees of cooking, gardening, and nutrition components. It is thus difficult to assess which aspect of these components and how intense (or what dose) these components were most likely to be associated with positive outcomes on children’s diet and nutrition related KAP. Besides, most of the SGBP did not consider the variation of teacher experience and motivation, which are some of the determinants for programme effectiveness. The use of varying teaching approaches and enthusiasm in curriculum delivery may influence students’ learning outcomes, and potentially determine the success of the intervention [54, 106]. In addition, the review might be prone to selection bias as only studies written in English were included. Furthermore, evidence was based on studies in which the participants were predominantly US and European populations with only a small amount from Asian or other countries, therefore the findings generated may not be generalizable and transferable to the other populations.

### Recommendations on future SGBP

To strengthen the impact of SGBP in promoting children’s KAP as observed from most of the successful interventions, integrated SGBP which include multiple or additional components such as nutritional education and parental involvement activities are encouraged to maximise the intervention effect. Classroom education is a crucial aspect of SGBP to effectively improve children’s nutritional-related knowledge. Thus, future SGBP is highly recommended to integrate with age-appropriate classroom education, conduct in smaller sample size with smaller child-to-staff ratio and shorter duration (~ 12 to 28 weeks), and intervene at an early age, preferably around the pre-school and primary school age, to achieve better outcomes on children’s nutritional-related knowledge and F&V consumption [107]. In addition, parental involvement in SGBP may help to promote children’s attitudes towards F&V, which may subsequently promote the intake of such foods.

Future SGBP should also adopt a multi-level approach, which covers the school, home and community environment to maximise the scope and therefore the impact of intervention. In addition, to further promote F&V consumption among children at school, it is recommended to incorporate food service into SGBP intervention. Potential ways include setting up a school salad bar in the cafeteria using the crops grown from SGBP or utilizing the produces to supplement the food in the cafeteria to increase the accessibility and availability of F&V at school. Future SGBP should also consider building relationships or partnerships with the local farmers or community gardens or, promoting or providing the students and their families the locally grown produce so as to maximise the exposure to F&V and the potential to promote such intake. Future studies should investigate the effective strategies to improve parental participation and involvement to strengthen the impact of SGBP in improving F&V consumption. In addition, it is essential for parents to acknowledge their key roles in shaping children’s eating habits. Future SGBP should offer more parental lessons and provide parents with the knowledge and tools to improve children’s eating behaviours [108]. This includes 1) offering practical advice on fostering children’s preferences towards healthier food options and increasing their willingness to consume unfamiliar food; 2) understanding the negative impact of coercive feeding practice and providing alternative options; 3) helping parents to establish a good parental role model; 4) educating the importance of not overfeeding their children and not forcing them to finish the meal when full [108]. Furthermore, more focus should be placed on investigating the long-term impact and the sustainability of the future SGBP.

## Conclusion

School garden-based programmes have generally shown beneficial effects on children’s knowledge of diet and nutrition, attitudes and acceptability towards vegetables with limited influence shown on dietary practices including the actual consumption of fruits and vegetables and the diversity of the diets. Impacts of SGBP on measured outcomes were highly influenced by various social and environmental factors with it being shown to be more effective when conducted at a younger age, for instance, in pre- or primary school-age children. In addition, positive outcomes found in children’s nutritional knowledge and dietary practices when conducted in a shorter intervention duration, and smaller sample size or smaller child-to-staff ratio, were possibly due to being more focused and accurately measured. Nevertheless, large heterogeneity was observed in the study design and methodologies, which have weakened the outcome significancy analysis. Parental involvement may help to better promote children’s attitudes, behaviours and willingness to consume fruits and vegetables. More measures are needed to be taken to encourage parental engagement so as to maximise the intervention effect. Future SGBP is suggested to use a combined multidisciplinary and multi-level approach targeting the children, parents and community to effectively promote healthy eating among the children and prevent childhood obesity. This would ensure that the interventions tackle individual intake as well as the factors affecting the social, family and school environment.

## Supplementary Information

**Additional file 1.**

## Data Availability

The datasets used and/or analysed during the current study are available from the corresponding author on reasonable request.

## Abbreviations

F&V: Fruits and vegetables
KAP: Knowledge, attitudes, and practices
SGBP: School garden-based programme(s)
UK: United Kingdom
US: United States
WHO: World Health Organization
