# Supplementary material for: Evaluating the impacts of school garden-based programmes on diet and nutrition-related knowledge, attitudes and practices among the school children: a systematic review
Source: BMC Public Health. 2022 Jun 24;22:1251. doi: 10.1186/s12889-022-13587-x (PMC9233338; doi:10.1186/s12889-022-13587-x)
Supplement: Supplementary file 1 — Additional file 1. [file 12889_2022_13587_MOESM1_ESM.docx]

**Supplementary table 1: Search strategies for each of the database**

**A. PubMed**

| **Steps** | **Search** | **Results** |
| --- | --- | --- |
| 1 | “child”[MeSH] OR “child, preschool” [MeSH] OR “students”[MeSH] OR “adolescent”[MeSH] | 1,742,170 |
| 2 | adolescen* [tiab] OR boy?[tiab] OR girl?[tiab] OR child*[tiab] OR children[tiab] OR juvenil*[tiab] OR kid?[tiab] OR preschool*[tiab] OR school*[tiab] OR teen*[tiab] OR youth*[tiab] OR young[tiab] OR "school children" [tiab] OR student*[tiab] | 1,663,099 |
| 3 | #1 OR #2 | 2,520,165 |
| 4 | "schools"[MeSH] OR "Schools, Nursery"[Mesh] OR school*[tiab] OR nurser* [tiab] OR kindergarten*[tiab] OR kindergarden*[tiab] | 253,422 |
| 5 | "gardens"[MeSH] OR "gardening"[MeSH] OR "Organic Agriculture"[MeSH] OR "food, organic" [MeSH] OR "fruit"[MeSH] OR "vegetables"[MeSH] OR "Plants"[Mesh] OR "Trees"[Mesh] OR "Seeds"[Mesh] OR garden*[tiab] OR gardening[tiab] OR plant*[tiab] OR fruit*[tiab] OR vegetable*[tiab] OR "fruit vegetable*"[tiab] OR "fruit growing"[tiab] OR "vegetable growing"[tiab] OR seed*[tiab] OR tree*[tiab] OR "organic agriculture"[tiab] OR "organic farming"[tiab] OR "organic food"[tiab] OR organic[tiab] OR farm[tiab] | 1,312,250 |
| 6 | #4 AND #5 | 10,627 |
| 7 | "appetite"[Mesh] OR "Diet"[Mesh] OR "diet, food, and nutrition"[Mesh] OR "Diet, Healthy"[Mesh] OR "health behavior"[MeSH] OR "food fussiness"[MeSH] OR "food preferences"[MeSH] OR "Attitude to Health"[Mesh] OR "health literacy"[MeSH] OR "health knowledge, attitudes, practice"[MeSH] OR "eating" [MeSH] | 1,045,590 |
| 8 | (eating[tiab] OR diet*[tiab] OR food[tiab] OR dietary[tiab] OR nutrition[tiab] OR nutritional[tiab] OR fruit*[tiab] OR vegetable*[tiab]) AND (knowledge[tiab] OR attitude*[tiab] OR practi?e*[tiab] OR behavio?r[tiab] OR preference*[tiab] OR habit*[tiab] OR intake*[tiab] OR consumption[tiab] OR healthy[tiab] OR literacy[tiab] OR skill*[tiab] or pattern*[tiab] OR diversity[tiab] OR diverse[tiab] OR perception*[tiab]) OR "energy intake"[tiab] OR "appetite"[tiab] OR "food fussiness"[tiab] OR "food neophobia"[tiab] | 375,571 |
| 9 | #7 or #8 | 1,231,854 |
| 10 | #3 AND #6 AND #9 | 5,088 |
| 11 | randomized controlled trial[pt] OR "randomized controlled trials as topic"[MeSH] OR "random allocation"[MeSH] | 572,843 |
| 12 | "follow-up studies"[MeSH] | 431,831 |
| 13 | "evaluation studies as topic"[MeSH] OR "program evaluation"[MeSH] OR "Controlled Before-After Studies"[MeSH] | 797,930 |
| 14 | #11 or #12 or #13 | 1,690,850 |
| 15 | “controlled trial*”[tiab] OR “intervention*”[tiab] OR randomised[tiab] OR randomized [tiab] OR trial*[tiab] OR "randomised controlled trial"[tiab] OR "randomized controlled trial"[tiab] | 1,797,829 |
| 16 | "follow-up stud*"[tiab] | 32,066 |
| 17 | "program evaluation*"[tiab] | 4,171 |
| 18 | #15 or #16 or #17 | 1,826,573 |
| 19 | #14 or #18 | 2,970,872 |
| 20 | #10 AND #19 | 2,190 |
|  | Apply filter (From Year 2000 to present) | 2,190 |

**B. Scopus**

| **Steps** | **Search** | **Results** |
| --- | --- | --- |
| 1 | TITLE-ABS-KEY (adolescen* OR boy? OR child* OR children OR girl? OR juvenil* OR kid? OR preschool* OR school* OR teen* OR youth* OR young OR "school children" OR student*) | 7,262,889 |
| 2 | TITLE-ABS-KEY (( school* OR nurser* OR kindergarten* OR kindergarden*) AND (garden* OR gardening OR plant* OR fruit* OR vegetable* OR "fruit vegetable*" OR "fruit growing" OR "vegetable growing" OR seed* OR tree* OR "organic agriculture" OR "organic farming" OR "organic food" OR organic OR farm )) | 46,314 |
| 3 | TITLE-ABS-KEY (( eating OR diet* OR food OR dietary OR nutrition OR nutritional OR fruit* OR vegetable*) AND (knowledge OR attitude OR practi?e* OR behaviou?r* OR preference* OR habit* OR intake* OR consumption* OR healthy OR skill* OR pattern* OR diversity OR diverse OR perception* )) | 1,193,444 |
| 4 | TITLE-ABS-KEY  ("intake" OR "appetite" OR "portion size" OR "food fussiness" OR "food neophobia") | 124,908 |
| 5 | #3 OR #4 | 1,248,399 |
| 6 | #1 AND #2 AND #5 | 9,219 |
| 7 | TITLE-ABS-KEY("randomized controlled trial*" OR "randomised controlled trail*" OR "random allocation*" OR "controlled trial*”OR “intervention*” OR randomised OR randomized OR trial*) | 3,904,284 |
| 8 | TITLE-ABS-KEY ("follow-up stud*") | 598,553 |
| 9 | TITLE-ABS-KEY ("program evaluation*" OR "controlled before-after stud*" ) | 72,830 |
| 10 | #6 OR #7 OR #8 | 3,953,105 |
| 11 | #6 AND #10 | 2,647 |
|  | limit to 2000 -2021 | 2,524 |

**C. Embase**

| **Search** | **Results** |
| --- | --- |
| 1. child/ or exp preschool child/ or exp school child/ | 1548775 |
| 2. (preschool child or preschool children or school child* or school children or child* or children or preschool*).tw. | 1457822 |
| 3. 1 or 2 | 2027230 |
| 4. student/ or exp elementary student/ or exp high school student/ or exp middle school student/ | 113525 |
| 5. (student* or elementary student* or high school student* or middle school student* or school*).tw. | 553833 |
| 6. 4 or 5 | 578131 |
| 7. exp adolescent/ or exp juvenile/ | 2666071 |
| 8. (adolescent* or teen* or juvenil* or youth* or young or kid?).tw. | 920054 |
| 9. 7 or 8 | 3091259 |
| 10. exp boy/ or exp girl/ | 54143 |
| 11. (boy? or girl?).tw. | 258281 |
| 12. 10 or 11 | 262569 |
| 13. 3 or 6 or 9 or 12 | 3738810 |
| 14. school/ or exp high school/ or exp middle school/ or exp primary school/ or exp kindergarden/ or exp kindergarten/ | 87758 |
| 15. (school* or high school* or middle school* or primary school* or kindergarden* or kindergarten* or nurser*).tw. | 312338 |
| 16. 14 or 15 | 321787 |
| 17. exp gardening/ | 1744 |
| 18. (gardening or garden*).tw. | 13427 |
| 19. 17 or 18 | 13894 |
| 20. plant/ or exp plant seed/ or exp "tree"/ | 201176 |
| 21. (plant* or plant seed* or seed* or tree*).tw. | 808470 |
| 22. 20 or 21 | 858541 |
| 23. exp fruit/ or exp fruit vegetable/ or exp fruit growing/ or exp vegetable/ | 275779 |
| 24. (fruit* or fruit vegetable* or fruit* growing or vegetable* or vegetable* growing).tw. | 154688 |
| 25. 23 or 24 | 346248 |
| 26. exp organic farming/ or exp organic food/ | 1964 |
| 27. (organic farming or organic agriculture or organic food or organic or farm).tw. | 336085 |
| 28. 26 or 27 | 336403 |
| 29. 19 or 22 or 25 or 28 | 1384577 |
| 30. exp attitude to health/ or exp nutritional health/ or exp health/ or exp health literacy/ or exp child nutrition/ or exp adolescent nutrition/ or exp nutrition/ | 2604274 |
| 31. (eating or diet* or food or dietary or nutrition or nutritional or fruit* or vegetable*).tw. | 1297698 |
| 32. (knowledge or attitude* or practi?e* or behavio?r or preference* or habit* or intake* or consumption* or healthy or literacy or skill* or pattern* or diversity or diverse or perception*).tw. | 5951242 |
| 33. 31 and 32 | 565506 |
| 34. 30 or 33 | 2742926 |
| 35. exp food fussiness/ or exp food-seeking behavior/ or exp food preference/ or exp food neophobia/ | 14696 |
| 36. (food fussiness or food neophobia).tw. | 446 |
| 37. 35 or 36 | 14936 |
| 38. exp appetite/ or exp eating habit/ | 32346 |
| 39. (appetite* or eating habit*).tw. | 45122 |
| 40. 38 or 39 | 58935 |
| 41. 34 or 37 or 40 | 2756955 |
| 42. 13 and 16 and 29 and 41 | 8128 |
| 43. exp randomized controlled trial/ | 599305 |
| 44. (randomi?ed controlled trial* or controlled trial* or intervention* or randomised or randomized or trial*).tw. | 2782977 |
| 45. 43 or 44 | 2906761 |
| 46. exp program evaluation/ | 26189 |
| 47. (program evaluation* or controlled before-after stud*).tw. | 4734 |
| 48. 46 or 47 | 29105 |
| 49. exp follow up/ | 1571493 |
| 50. follow-up.tw. | 1461648 |
| 51. 49 or 50 | 1894925 |
| 52. 45 or 48 or 51 | 4375304 |
| 53. 42 and 52 | 2859 |
| limit to 2000 - current | 2801 |

**D. Cochrane Library**

| **Steps** | **Search** | **Results** |
| --- | --- | --- |
| #1 | MeSH descriptor: [Child] explode all trees | 56347 |
| #2 | MeSH descriptor: [Child, Preschool] explode all trees | 29652 |
| #3 | MeSH descriptor: [Adolescent] explode all trees | 104274 |
| #4 | #1 OR #2 OR #3 | 131989 |
| #5 | (adolescen* OR boy? OR child* OR children OR girl? OR juvenil* OR kid? OR preschool* OR school* OR teen* OR youth* OR young OR "school children" OR student*):ti,ab,kw | 339293 |
| #6 | #4 OR #5 | 339293 |
| #7 | MeSH descriptor: [Schools] explode all trees | 3075 |
| #8 | (school* OR nurser* OR kindergarten* OR kindergarden*):ti,ab,kw | 36315 |
| #9 | #7 OR #8 | 37177 |
| #10 | MeSH descriptor: [Gardens] explode all trees | 7 |
| #11 | MeSH descriptor: [Gardening] explode all trees | 33 |
| #12 | MeSH descriptor: [Organic Agriculture] explode all trees | 1 |
| #13 | MeSH descriptor: [Food, Organic] explode all trees | 53 |
| #14 | MeSH descriptor: [Plants] explode all trees | 11735 |
| #15 | MeSH descriptor: [Fruit] explode all trees | 2637 |
| #16 | MeSH descriptor: [Vegetables] explode all trees | 1879 |
| #17 | MeSH descriptor: [Seeds] explode all trees | 931 |
| #18 | MeSH descriptor: [Trees] explode all trees | 84 |
| #19 | (garden* OR gardening OR plant* OR fruit* OR vegetable* OR "fruit vegetable*" OR "fruit growing" OR "vegetable growing" OR seed* OR tree* OR "organic agriculture" OR "organic farming" OR "organic food" OR organic OR farm):ti,ab,kw | 35230 |
| #20 | #10 OR #11 OR #12 OR #13 OR #14 OR #15 OR #16 OR #17 OR #18 OR #19 | 39901 |
| #21 | #9 AND #20 | 1597 |
| #22 | MeSH descriptor: [Intake] explode all trees | 5388 |
| #23 | MeSH descriptor: [Appetite] explode all trees | 1456 |
| #24 | MeSH descriptor: [Diet] explode all trees | 18515 |
| #25 | MeSH descriptor: [Diet, Food, and Nutrition] explode all trees | 53990 |
| #26 | MeSH descriptor: [Health Behavior] explode all trees | 35906 |
| #27 | MeSH descriptor: [Food Fussiness] explode all trees | 4 |
| #28 | MeSH descriptor: [Food Preferences] explode all trees | 882 |
| #29 | MeSH descriptor: [Attitude to Health] explode all trees | 35652 |
| #30 | MeSH descriptor: [Health Literacy] explode all trees | 384 |
| #31 | MeSH descriptor: [Health Knowledge, Attitudes, Practice] explode all trees | 5993 |
| #32 | MeSH descriptor: [Eating] explode all trees | 3573 |
| #33 | ((eating OR diet* OR food OR dietary OR nutrition OR nutritional OR fruit* OR vegetable*) AND (knowledge OR attitude OR practi?e* OR behaviour?r* OR preference* OR habit* OR intake* OR consumption* OR healthy OR skill* OR pattern* OR diversity OR diverse OR perception* ) OR "energy intake"  OR  "appetite"  OR  "food fussiness"  OR  "food neophobia"):ti,ab,kw | 85216 |
| #34 | #22 OR #23 OR #24 OR #25 OR #26 OR #27 OR #28 OR #29 OR #30 OR #31 OR #32 OR #33 | 147205 |
| #35 | #6 AND #21 AND #34 | 1111 |
| #36 | MeSH descriptor: [Random Allocation] explode all trees | 20611 |
| #37 | MeSH descriptor: [Randomized Controlled Trial] explode all trees | 119 |
| #38 | MeSH descriptor: [Randomized Controlled Trials as Topic] explode all trees | 14687 |
| #39 | ("controlled trial*" OR "intervention*" OR "randomised" OR "randomized" OR "trial*" OR "randomised controlled trial*" OR "randomized controlled trial*"):ti,ab,kw | 1183375 |
| #40 | #36 OR #37 OR #38 OR #39 | 1190448 |
| #41 | MeSH descriptor: [Follow-Up Studies] explode all trees | 59297 |
| #42 | ("follow up studies" OR "follow up study"):ti,ab,kw | 63570 |
| #43 | #41 OR #42 | 63570 |
| #44 | MeSH descriptor: [Program Evaluation] explode all trees | 6261 |
| #45 | MeSH descriptor: [Evaluation Studies as Topic] explode all trees | 51062 |
| #46 | MeSH descriptor: [Controlled Before-After Studies] explode all trees | 71 |
| #47 | ("program evaluation*"):ti,ab,kw | 7440 |
| #48 | #44 OR #45 OR #46 OR #47 | 52458 |
| #49 | #40 OR #43 OR #48 | 1220316 |
| #50 | #35 AND #49 | 1032 |
|  | limit to 2000 -2021 | 1032 |

**E. Web of Science**

| **Steps** | **Search** | **Results** |
| --- | --- | --- |
| #1 | TS=(adolescen* OR boy? OR child* OR children OR girl? OR juvenil* OR kid? OR preschool* OR school* OR teen* OR youth* OR young OR "school children" OR student*) | 3,170,235 |
| # 2 | TS=(school* OR nurser* OR kindergarten* OR kindergarden*) | 545,546 |
| # 3 | TS=(garden* OR gardening OR plant* OR fruit* OR vegetable* OR "fruit vegetable*" OR "fruit growing" OR "vegetable growing" OR seed* OR tree* OR "organic agriculture" OR "organic farming" OR "organic food" OR organic OR farm) | 3,143,637 |
| # 4 | #2 AND #3 | 25,881 |
| # 5 | TS=((eating OR diet* OR food OR dietary OR nutrition OR nutritional OR fruit* OR vegetable*) AND (knowledge OR attitude OR practi?e* OR behaviour?r* OR preference* OR habit* OR intake* OR consumption* OR healthy OR skill* OR pattern* OR diversity OR diverse OR perception*) OR "energy intake"  OR  "appetite" OR  "food fussiness"  OR  "food neophobia") | 696,741 |
| # 6 | #1 AND #4 AND #5 | 6,396 |
| # 7 | TS=(“controlled trial*” OR “intervention*” OR randomised OR randomized OR trial*OR "randomised controlled trial*"OR "randomized controlled trial*") | 1,793,713 |
| # 8 | TS=("follow-up stud*") | 39,054 |
| # 9 | TS=("program evaluation*" OR "controlled before-after stud*") | 7,647 |
| # 10 | #7 OR #8 OR #9 | 1,831,456 |
| # 11 | #6 AND #10 | 2,289 |
|  | limit to 2000 - 2021 | 2,289 |

**Supplementary Table 2: Quality appraisal of included studies**

| **Author (Year) and country** | **Validity questions** | | | | | | | | | | |
| --- | --- | --- | --- | --- | --- | --- | --- | --- | --- | --- | --- |
|  | **Research question** | **No selection bias** | **Randomisation/ group comparability** | **Withdrawal description** | **Blinding** | **Procedure’ description** | **Outcome’s description** | **Statistical analysis** | **Results support conclusion** | **No funding or sponsorship bias** | **Overall** |
| Barnard et al., (2020) USA | Y | Y | N | N | N | Y | Y | N | Y | Y | ∅ |
| Bontrager Yoder et al., (2014) USA | Y | Y | N | N | N | Y | Y | N | Y | U | ∅ |
| Cotter et al., (2013) Portugal | Y | Y | Y | N | N | Y | Y | N | Y | Y | + |
| Davis et al., (2021) USA | Y | Y | Y | N | Y | Y | Y | N | Y | Y | + |
| Davis et al., (2016) USA | Y | Y | Y | Y | N | Y | Y | N | Y | Y | + |
| Davis et al., (2011) USA | Y | Y | N | N | N | Y | Y | N | Y | Y | ∅ |
| Duncan et al., (2015) UK | Y | U | U | N | N | Y | y | N | Y | U | ∅ |
| Gatto et al., (2017) USA | Y | Y | Y | Y | N | Y | Y | N | Y | Y | + |
| Gatto et al., (2012) USA | Y | Y | Y | N | N | Y | Y | N | Y | Y | + |
| Gibbs et al., (2013) Australia | Y | Y | Y | Y | N | Y | Y | Y | Y | N | + |
| Hanbazaza et al., (2015) Canada | Y | N | N | N | N | Y | Y | N | Y | Y | ∅ |
| Jaenke et al., (2012) Australia | Y | Y | N | N | N | Y | Y | N | Y | Y | ∅ |
| Khan et al., (2019) UK | Y | Y | Y | N | N | Y | Y | N | Y | Y | + |
| Kim et al., (2020) South Korea | Y | Y | N | N | N | Y | Y | N | N | Y | ∅ |
| Landry et al., (2019) USA | Y | Y | Y | Y | N | Y | Y | N | Y | Y | + |
| Lee et al., (2017) USA | Y | Y | N | N | N | Y | Y | N | Y | Y | ∅ |
| Leuven et al., (2018) Netherlands | Y | Y | N | N | N | Y | Y | N | Y | Y | ∅ |
| Massarani et al., (2019) Rio de Janeiro | Y | Y | N | Y | N | Y | Y | N | Y | Y | ∅ |
| McAleese et al., (2007) USA | Y | Y | Y | Y | N | Y | Y | N | Y | U | + |
| Morgan et al. (2010) Australia | Y | Y | N | Y | N | Y | Y | N | Y | Y | ∅ |
| Nele Huys et al., (2019) Ghent | y | Y | Y | Y | N | Y | Y | N | Y | U | + |
| Parmer et al., (2009) USA | Y | N | N | N | N | Y | Y | N | Y | U | ∅ |
| Ratcliffe et al., (2011) USA | Y | N | N | N | N | Y | Y | N | Y | U | ∅ |
| Schreinemachers et al., (2020) Nepal | Y | Y | Y | N | N | Y | Y | N | Y | Y | + |
| Schreinemachers et al., (2019) Burkina Faso | Y | Y | Y | N | N | Y | Y | N | Y | Y | + |
| Schreinemachers et al., (2017) Bhutan | Y | Y | Y | N | N | Y | Y | N | Y | Y | + |
| Schreinemachers et al., (2017) Nepal | Y | Y | Y | Y | N | Y | Y | Y | Y | Y | + |
| Sharma et al., (2015) USA | Y | Y | N | N | N | Y | Y | N | Y | Y | ∅ |
| Shrestha et al., (2020) Nepal | Y | Y | Y | Y | Y | Y | Y | Y | Y | U | + |
| Somerset et al., (2009) Australia | Y | Y | N | N | N | Y | Y | N | Y | Y | ∅ |
| Spears-Lanoix et al., (2015) USA | Y | Y | N | N | N | Y | Y | N | Y | U | ∅ |
| Taylor et al., (2018) USA | Y | Y | Y | N | Y | Y | Y | N | Y | N | + |
| van den Berg et al., (2020) USA | Y | Y | Y | N | N | Y | Y | N | Y | Y | + |
| Wells et al., (2018) USA | Y | Y | Y | N | N | Y | U | N | Y | Y | ∅ |
| Wells et al., (2015) USA | Y | Y | Y | Y | N | Y | Y | N | Y | Y | + |

Y = Yes

N = No

U = Unclear

+ = Low risk of bias

∅ = Unlcear risk of bias
